# MAGOH and MAGOHB Knockdown in Melanoma Cells Decreases Nonsense-Mediated Decay Activity and Promotes Apoptosis via Upregulation of GADD45A

**DOI:** 10.3390/cells11233859

**Published:** 2022-11-30

**Authors:** Agnes Soederberg, Tina Meißgeier, Anja Katrin Bosserhoff, Lisa Linck-Paulus

**Affiliations:** 1 Institute of Biochemistry, Friedrich-Alexander-Universität Erlangen-Nürnberg (FAU), Fahrstraße 17, 91054 Erlangen, Germany; 2 Comprehensive Cancer Center (CCC) Erlangen-EMN, 91054 Erlangen, Germany

**Keywords:** MAGOH, exon junction complex, nonsense-mediated decay, GADD45A, melanoma

## Abstract

Cutaneous malignant melanoma is a highly proliferative and aggressive skin cancer with a steadily increasing incidence and a low long-term survival rate after metastatic progression. The protein MAGOH and its highly identical homologue MAGOHB are core components of the exon junction complex (EJC), which regulates splicing, stability and translation of mRNAs. The EJC, and especially MAGOH, has been shown to be involved in the development and progression of several cancers. In melanoma, the expression and function of both homologues remain essentially unexplored. This study identifies high MAGOH and MAGOHB protein expression in cutaneous melanoma cell lines and patient derived tissue samples. An siRNA-mediated knockdown of MAGOH significantly inhibits melanoma cell proliferation. The loss of MAGOH does not affect cell cycle progression, but induces apoptosis, an effect that is enhanced by a simultaneous knockdown of MAGOH and MAGOHB. MAGOH and MAGOHB do not influence the expression of the pro-apoptotic protein Bcl-XS or exon skipping. However, the knockdown of MAGOH and MAGOHB strongly decreases nonsense-mediated decay (NMD) activity, leading to an upregulation of the pro-apoptotic protein GADD45A. In conclusion, simultaneous inhibition of MAGOH and MAGOHB expression substantially affects cell survival, indicating both MAGOH homologues as promising new targets for the treatment of melanoma.

## 1. Introduction

Cutaneous malignant melanoma is the deadliest form of skin cancer [1]. It is characterized by a wide degree of heterogeneity in terms of clinical and histopathological appearance [2,3], as well as genomic variability [4,5,6], which makes this disease a significant public health problem. It has had a steadily increasing incidence over the last 50 years and is currently the sixth most common cancer in European countries [7,8]. Cutaneous melanoma originates from a malignant transformation of melanocytes, the pigment producing cells in the skin, driven by genetic alterations most commonly caused by UV-radiation from sun exposure [9]. The most important prognostic factor for melanoma is tumor thickness and depth (Breslow classification), followed by ulceration [9]. Further, recent evidence has shown that the relationship between melanoma thickness or mitotic index and time to diagnosis is a strong prognostic factor [10,11]. Despite major advancements in precision medicine and immunotherapy over the last decade, the long-term survival rate of metastatic melanoma of the skin remains low [12,13]. This emphasizes the current need to identify novel therapeutic targets for the treatment of malignant melanoma.

The exon junction complex (EJC) is an RNA-binding protein complex, which is involved in post-transcriptional regulation of gene expression, thereby affecting almost all important cellular pathways [14]. It is deposited on the precursor messenger RNA (pre-mRNA) during splicing, upstream from newly formed exon-exon junctions. On the one hand, the EJC can improve the translation efficiency of its bound mRNA by recruiting factors that enhance translation initiation [15]. On the other hand, the EJC can inhibit translation by inducing nonsense-mediated decay (NMD) of incorrectly assembled mRNAs [15]. The process of NMD is initiated when a premature termination codon (PTC), which can arise due to aberrant mRNA splicing or frameshift mutations, is located upstream of an exon-exon junction. In this case, the EJC remains bound downstream of the PTC and is sensed by additional factors that mediate the degradation of the mRNA [15]. NMD is an especially essential mechanism during tumorigenesis, as genomic instability can lead to a toxic increase in PTC-containing mRNA transcripts [16]. Tumor cells have been suggested to counterbalance this effect through an upregulation of EJC components [17]. Another essential function of the EJC is its role in regulating alternative splicing. By interacting with the spliceosome, the EJC can control the use of alternative splice sites and prevent erroneous splicing of cryptic splice sites in coding exon-exon junctions [17,18,19].

The core of the human EJC consist of the “RNA Binding Motif Protein 8A” (RBM8A), the “Eukaryotic Translation Initiation Factor 4A3” (EIF4A3) and the human “Mago-Nashi Homolog” (MAGOH). Humans have two homologues of the MAGOH protein, MAGOH and MAGOHB, which are located on two different chromosomes and share 87% DNA sequence identity [20]. On the protein level, MAGOH and MAGOHB only differ in the first four amino acids and they have been demonstrated to form the EJC with the same level of affinity, indicating a functional redundancy [20].

A role of MAGOH for tumorigenesis has been described in some cancers [21,22,23]. In gastric cancer, both MAGOH and MAGOHB have been identified to regulate key pathological processes such as cell proliferation and cell cycle progression [24]. In addition, MAGOH has been demonstrated as an important regulator of melanocyte development in the skin [25]. In a study by Silver et al. it was discovered that MAGOH haploinsufficient mice exhibited a reduced number of epidermal melanoblasts during embryogenesis and displayed a hypopigmented phenotype as adults [25]. It was further shown that MAGOH depletion in a melanoma cell line causes growth arrest. However, the molecular mechanisms of MAGOH in melanoma pathogenesis as well as the importance of MAGOHB in this process has so far not been investigated.

This study aims to examine the expression of the homologues MAGOH and MAGOHB in melanocytes, several cutaneous melanoma cell lines and patient derived tissue samples and further elucidates the molecular function of both homologues in melanoma.

## 2. Materials and Methods

### 2.1. Cultivation of Melanoma Cell Lines, Fibroblasts and Melanocytes

Human cutaneous melanoma cell lines (Table 1) were cultivated in “Dulbecco’s Modified Eagle’s Medium” (DMEM) (Sigma-Aldrich Chemie GmbH, Steinheim, Germany), “RPMI-1640 Medium” (Sigma-Aldrich Chemie GmbH, Steinheim, Germany) or tumor medium (TU: 80% MCDB152 (Sigma-Aldrich Chemie GmbH, Steinheim, Germany), 20% Leibovitz L15 (Sigma-Aldrich Chemie GmbH, Steinheim, Germany), 5 µg/mL Insulin, 1.68 mM CaCl_2_). All media were supplemented with fetal bovine serum (FBS) (Sigma-Aldrich Chemie GmbH, Steinheim, Germany) (DMEM and RPMI 10%, TU 2%), 100 U/mL penicillin and 0.1 mg/mL streptomycin (Sigma-Aldrich Chemie GmbH, Steinheim, Germany). The RPMI-1640 medium was additionally supplemented with 0.2% sodium bicarbonate. Human fibroblasts (BJ1) were kindly provided by Dr. Ingo Thievessen (Biophysics, Center for Medicine, Physics and Technology, FAU Erlangen-Nürnberg, Erlangen, Germany) and cultivated in DMEM high glucose (4500 mg/L) medium (Sigma-Aldrich Chemie GmbH, Steinheim, Germany). Normal human epidermal melanocytes (NHEM) were obtained from PromoCell (Heidelberg, Germany) and grown in “Melanocyte Growth Medium M2” without phorbol myristate acetate (PromoCell, Heidelberg, Germany). Cells were cultivated at 37 °C in a humidified atmosphere with either 8% or 5% CO_2_ and split every 2–3 days via trypsinization.

### 2.2. SiRNA-Mediated Knockdown (KD)

To knockdown the expression of MAGOH, MAGOHB and GADD45A, melanoma cells were transfected with single siRNAs (siMAGOH, siMAGOH2, siMAGOHB, siGADD45A) or with a siPool constituting a mixture of 30 siRNAs targeting both MAGOH and MAGOHB (Table 2). Transfection was carried out in six-well plates using 5 µL “Lipofectamine RNAiMAX” (Invitrogen, Thermo Fischer Scientific, Waltham, MA, USA) in medium without phenol red (PAN-Biotech GmbH, Aidenbach, Germany) and without FBS and with final siRNA concentrations of 50 nM (single siRNA) or 2.5 nM (siPool). 

### 2.3. Western Blot

Western blot analysis was performed as described previously [27], with the use of the following antibodies: MAGOH (clone 21B12, sc-56724 (Santa Cruz Biotechnology, Dallas, TX, USA) 1:1000 in 5% BSA/TBS-T), β-Actin (cloneAC15/A5441 (Sigma-Aldrich Chemie GmbH, Steinheim, Germany) 1:5.000 in PBS) and anti-mouse-HRP (Cell Signaling Technology Inc., Danvers, MA, USA) 1:3000 in TBS-T). Quantification of the Western blots was carried out using LabImage 1D (Kapelan Bio-Imaging GmbH, Leipzig, Germany).

### 2.4. Quantitative Real Time Polymerase Chain Reaction (qRT-PCR)

RNA extraction, cDNA synthesis and qRT-PCR was performed as described before [27], using the primer sequences listed in Table 3. Relative mRNA expression of the target genes was analyzed compared to the housekeeping gene β-actin using the ΔCP method and normalized to a control; for a direct comparison of the expression of different genes, primer efficiency was determined.

### 2.5. Cell Proliferation Assay

Cell proliferation was measured using the “Cell Proliferation Kit II (XTT)” (Roche Diagnostics GmbH, Mannheim, Germany). After 24 h of siRNA transfection, the cells were seeded in triplicates (500 cells/well) in 96-well plates in medium without phenol red (PAN-Biotech GmbH, Aidenbach, Germany) and quantification of cell viability was carried out according to the manufacturer’s instructions after 1, 4, 5, 6 and 7 days. The absorbance at 490 nm was measured with a “CLARIOStar” plate reader (BMG Labtech GmbH, Ortenberg, Germany), after four hours of incubation with the XTT reagents.

### 2.6. Clonogenic Assay

After 24 h of siRNA transfection, the cells were seeded in six-well plates in a low cell number (500 cells/well) and left for 7–10 days to form colonies before staining and fixation with 6% glutaraldehyde and 0.36% crystal violet, for 30 min at room temperature. The number of colonies in each well was analyzed from scanned pictures of the plates with the “CellSens Dimension” software (Olympus K.K., Shinjuku, Tokyo, Japan).

### 2.7. Cell Cycle Analysis

Cell cycle analysis was performed 96 h after siRNA transfection as described previously [28]. The data was analyzed with the “FlowJo_v10.8.0” software (Becton, Dickinson and Company, Franklin Lakes, NJ, USA) and after gating of single cells the “Dean Jett Fox” algorithm [29] was used to distinguish the cell cycle phases.

### 2.8. Apoptosis Assay

For apoptosis analysis, siRNA-mediated KD was performed for 96 h before both the attached and non-attached cells were collected and stained using the “Annexin V-FITC Detection Kit” (PromoKine, PromoCell GmbH, Heidelberg, Germany) according to the manufacturer’s instructions. The samples were analyzed with FACS (“LSRFortessaTM”, BD Biosciences, Franklin Lakes, NJ, USA) and data interpretation was carried out with “BD FACSDiva 9.0” software (BD Biosciences, Franklin Lakes, NJ, USA).

### 2.9. Analysis of Exon Skipping

Following 24 h of siRNA-mediated KD, the green fluorescent (FRE5) control plasmid, the red fluorescent (FRE5Cf) control plasmid or the splicing reporter plasmid (RG6), gifts from Thomas Cooper (Addgene plasmid #80167; http://n2t.net/addgene:80167 (accessed on 26 September 2022); RRID:lAddgene_80167, Addgene plasmid #62377; http://n2t.net/addgene:62377 (accessed on 26 September 2022); RRID:Addgene_62377 and Addgene plasmid #62378 http://n2t.net/addgene:62378 (accessed on 26 September 2022); RRID:Addgene_62378) all previously described [30], were transfected to the cells using the “Lipofectamine LTX-Transfection Kit”(Invitrogen, Thermo Fischer Scientific, Waltham, MA, USA). Cells transfected with an empty pcDNA3.1(+) vector (GeneArt, Thermo Fischer Scientific, Waltham, MA, USA) were used as a negative control. Plasmid transfection was conducted by mixing 1 µg plasmid DNA to a total volume of 17 µL medium without phenol red (PAN-Biotech GmbH, Aidenbach, Germany), followed by the addition of 3 µL Plus Reagent. The LTX was prepared by adding 2.5 µL Lipofectamine LTX to 17.5 µL DMEM transfection medium. After 10 min incubation, the two were mixed 1:1 and incubated for an additional 30 min. Medium was exchanged for the cells and 40 µL of the lipofectamine-plasmid complexes was added to each well of a six-well plate. After 24 h, the cells were analyzed with flow cytometry (“LSRFortessaTM”, BD Biosciences, Franklin Lakes, NJ, USA) and the data interpreted with the “BD FACSDiva 9.0” software (BD Biosciences, Franklin Lakes, NJ, USA).

### 2.10. Analysis of Nonsense-Mediated Decay (NMD) Activity

NMD pathway activity was investigated 24 h after siRNA transfection by transfection of the NMD reporter vector (pKC4.06; gift from James Inglese, Addgene plasmid #112084; http://n2t.net/addgene:112084 (accessed on 26 September 2022); RRID:Addgene_112084). This CMV promoter driven vector encodes a firefly luciferase and contains a premature termination codon (PTC). As a control, the same vector construct without a PTC was used (pKC4.04.; gift from James Inglese, Addgene plasmid #112085; http://n2t.net/addgene:112085 (accessed on 26 September 2022); RRID:Addgene112085). Both vectors have previously been described [31]. Transfection was carried out in the same manner as described in the section “Analysis of Exon Skipping”, with a concentration of 0.5 µg vector DNA/well. For this experiment, the cells were also co-transfected with 22 ng of a TK promoter driven Renilla luciferase vector (pRL-TK; Promega, Madison, WI, USA), which was additionally added to the Lipofectamine Plus mixture. The relative light unit (RLU) was analyzed 24 h after transfection using the “Dual-Luciferase Reporter Assay System” (Promega, Madison, WI, USA), according to the manufacturers’ instructions, and measured with a “Berthold Centro LB 960” luminometer (Berthold Technologies, Bad Wildbad, Germany). For each sample, the NMD activity was calculated according to the following equation, as described in [32]:

$$\mathrm{NMD}\;\mathrm{activity}=\frac{\mathrm{Average}\;\left(\mathrm{pKC}4.06\;\mathrm{Firefly}/\mathrm{Renilla}\right)\;}{\mathrm{Average}\;\left(\mathrm{pKC}4.04\;\mathrm{Firefly}/\mathrm{Renilla}\right)\;}$$

### 2.11. Immunohistochemistry

Immunohistochemical analysis of patient-derived human cutaneous malignant melanoma tissue samples was performed as described previously [33]. Therefore, formalin fixed, paraffin embedded human cutaneous melanoma biopsies were microdissected (5 µm) and stained with a MAGOH/MAGOHB antibody using a 1:50 dilution (sc-56724, Santa Cruz Biotechnology, Dallas, TX, USA). Pictures were taken using the microscope “IX83” (Olympus K.K., Shinjuku, Tokyo, Japan) with 20× magnification. Seven human cutaneous melanoma samples derived from primary tumors and five metastasis-derived samples were analyzed. 

### 2.12. Statistical Analysis

Statistical significance was determined using the “GraphPad Prism 5.0.4.533” software (GraphPad Software Inc., San Diego, CA, USA). The number of biological replicates (n) included in the calculation of significance is indicated in the figure legend. For comparison of the two groups, an unpaired *t*-test was performed. For comparison of values normalized to the respective control, where the control was set as 1, one sample *t*-test was used. Three or more groups were compared using one-way analysis of variance (ANOVA) and subsequent Tukey’s Multiple Comparison Test.

## 3. Results

### 3.1. MAGOH mRNA Shows Higher Expression Than MAGOHB in Cutaneous Melanoma

**Figure 1:**
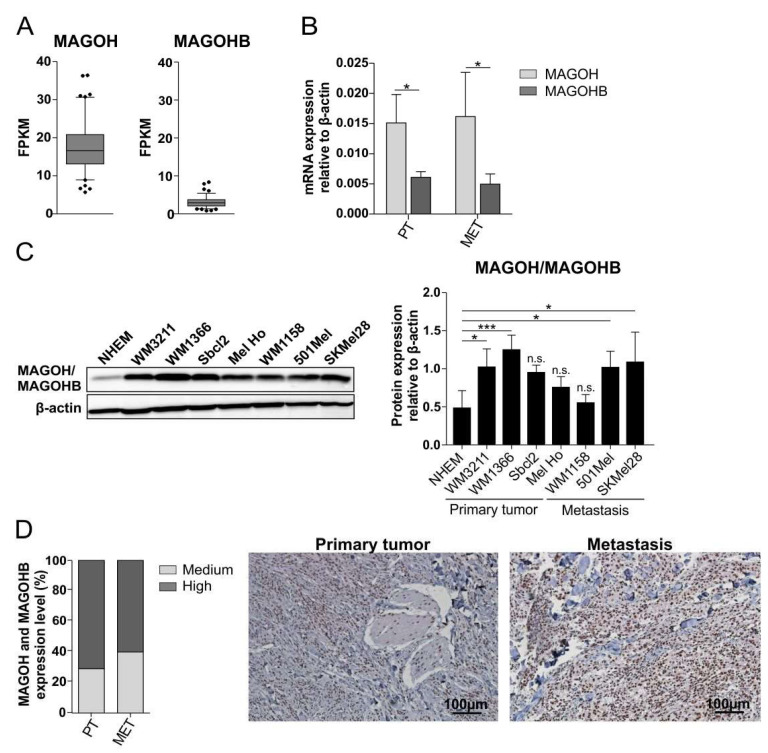
MAGOH mRNA is predominantly expressed compared to MAGOHB in cutaneous melanoma patients and tumor derived cutaneous melanoma cell lines and MAGOH/MAGOHB protein levels are enhanced in melanoma cells and skin melanoma derived tissue samples. (**A**) Fragments per kilobase of transcript per million mapped reads (FPKM) of RNA-sequencing data of cutaneous malignant melanoma patients from TCGA obtained from “The Human Protein Atlas” for MAGOH and MAGOHB mRNA (box and whiskers show mean and 5–95 percentile, outliers are shown as dots). (**B**) Relative MAGOH and MAGOHB mRNA expression compared to β-actin (ΔCP), measured with qRT-PCR, in cutaneous melanoma cell lines derived from primary tumors (PT) or metastases (MET) (mean ± SD from 4 PT and 5 MET cell lines, expression was measured for each cell line in three independent experiments, * = *p* < 0.05, unpaired *t*-test). (**C**) Protein expression of MAGOH and MAGOHB in indicated cell lines, analyzed with Western blot. One representative image is shown. Bar graph shows the quantification of MAGOH/MAGOHB protein levels compared to β-actin (mean ± SD of n = 3 (NHEM, WM1132, Sbcl2) or n = 4 (WM1366, Mel Ho, WM1158, 501Mel, SKMel28), * = *p* < 0.05, *** = *p* < 0.001, n.s. = not significant, one-way ANOVA with Tukey’s Multiple Comparison Test). (**D**) Immunohistochemical analysis of human patient derived tumor samples from cutaneous malignant melanoma primary tumors (PT) and metastases (MET) stained for MAGOH/MAGOHB protein expression. The graph represents the evaluation of 7 PT and 5 MET samples, pictures show two representative tumors.

To analyze the role of MAGOH and MAGOHB expression for tumorigenesis of cutaneous malignant melanoma, we examined RNA-sequencing (RNA-seq) data from “The Cancer Genome Atlas” (TCGA) regarding MAGOH and MAGOHB mRNA expression in cutaneous melanoma patients. The data show a substantially higher mRNA expression of MAGOH than MAGOHB: mean fragments per kilobase of transcript per million mapped reads (FPKM) for MAGOH were 16.58 compared to 2.88 for MAGOHB (Figure 1A) [34,35,36]. 

We further analyzed MAGOH and MAGOHB mRNA expression in different cutaneous melanoma cell lines derived from primary tumors (PT) or metastases (MET) using qRT-PCR and also observed a significantly higher expression of MAGOH compared to MAGOHB (Figure 1B).

### 3.2. MAGOH/MAGOHB Protein Expression Is Upregulated in Cutaneous Melanoma Cell Lines and Tissue Samples 

To analyze the protein expression of MAGOH and MAGOHB in melanoma, we performed Western blot experiments of several cutaneous melanoma cell lines as well as from normal human epidermal melanocytes (NHEM). As a result of the high sequence identity of MAGOH and MAGOHB at the protein level, all available antibodies detect both homologues, meaning that the Western blots reflect the combined expression of both MAGOH and MAGOHB. We observed an increased MAGOH/MAGOHB protein expression in most melanoma cell lines compared to healthy NHEM (Figure 1C). To further investigate MAGOH/MAGOHB expression at the protein level and its significance for melanoma development we performed immunohistochemical analysis of patient derived human tissue samples of primary or metastasis derived cutaneous malignant melanomas. We observed that MAGOH/MAGOHB is highly expressed and mostly located in the cell nuclei in all cutaneous melanoma tumor samples (Figure 1D).

### 3.3. An siRNA-Mediated Knockdown (KD) of MAGOH and MAGOHB Proves MAGOH to Be the Predominatnly Expressed Protein of Both Homologues in Cutaneous Melanoma Cells

**Figure 2:**
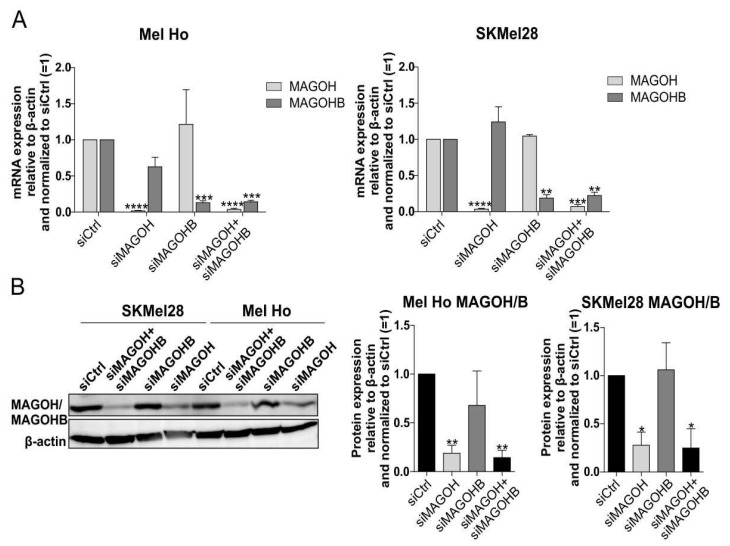
SiRNA-mediated knockdown of MAGOH and MAGOHB expression in the cutaneous melanoma cell lines Mel Ho and SKMel28. (**A**) Relative MAGOH and MAGOHB mRNA expression compared to β-actin(ΔCP), measured with qRT-PCR, after 24 h (Mel Ho) or 48 h (SKMel28) of transfection with siRNAs targeting MAGOH, MAGOHB or both, normalized to a control siRNA (mean ± SD of n = 3, ** = *p* < 0.01, *** = *p* < 0.001, **** = *p* < 0.0001, one sample *t*-test compared to 1). (**B**) Western blot analysis of MAGOH/MAGOHB protein expression after 48 h transfection of Mel Ho and SKMel28 with siRNAs targeting MAGOH, MAGOHB or both, compared to a control siRNA. Bar graphs show the quantification of MAGOH/MAGOHB protein levels compared to β-actin (mean ± SD from n = 3, * = *p* < 0.05, ** = *p* < 0.01, one sample *t*-test compared to 1).

Our data indicate a critical role of MAGOH/MAGOHB expression for the tumor development of cutaneous malignant melanoma. To analyze which role each of the two homologues plays for melanoma tumorigenesis, siRNA-mediated knockdown (KD) of MAGOH and MAGOHB was performed for two cutaneous melanoma cell lines, Mel Ho and SKMel28, in a single and combined approach. The KD was proven efficient on the RNA level with the use of qRT-PCR as a KD of MAGOH (siMAGOH) significantly reduced MAGOH mRNA expression and a KD of MAGOHB (siMAGOHB) significantly reduced MAGOHB (Figure 2A). The combined KD of both (siMAGOH + siMAGOHB) significantly reduced the mRNA expression of both homologues to a comparable level with the respective single siRNA. 

Western blot experiments showed a significantly reduced MAGOH/MAGOHB protein expression after single MAGOH and after the combined MAGOH and MAGOHB KD (Figure 2B). The single KD of MAGOHB did not strongly reduce the total MAGOH/MAGOHB protein levels. As the KD of MAGOHB had proven to be efficient on the mRNA level (Figure 2A), the absence of a KD of MAGOHB on the protein level supports the conclusion that MAGOH accounts for the majority of the total MAGOH and MAGOHB protein expression in cutaneous melanoma.

### 3.4. MAGOH Expression Is Vital for Melanoma Cell Proliferation

**Figure 3:**
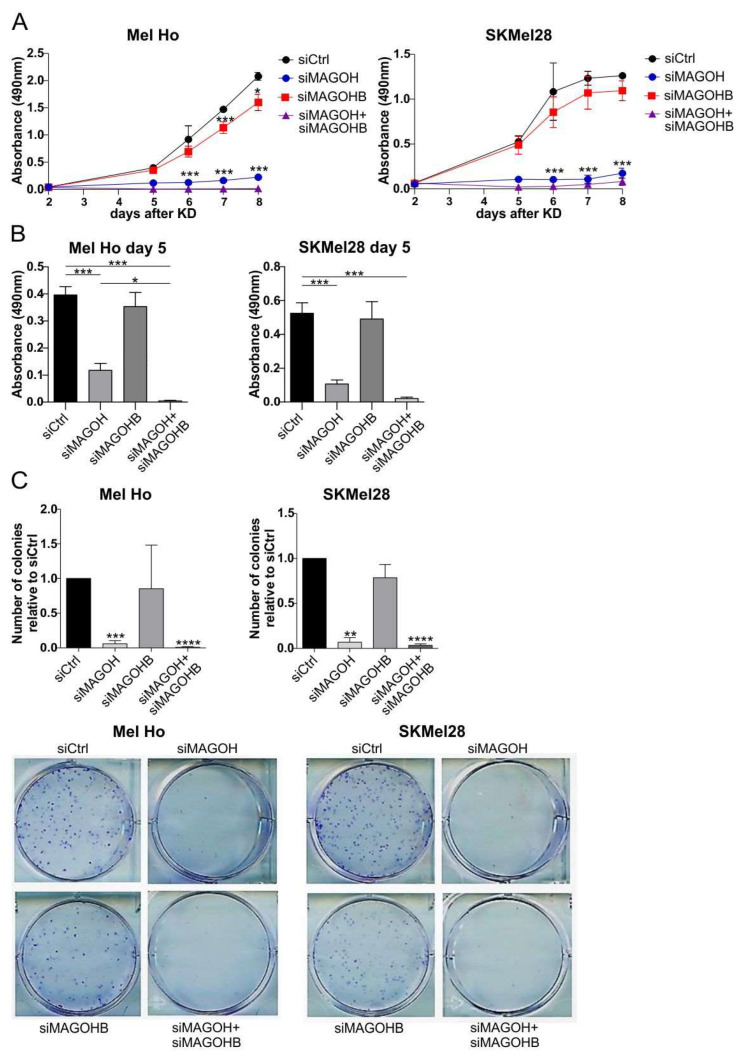
Knockdown of MAGOH, as well as of MAGOH and MAGOHB significantly inhibits cutaneous melanoma cell proliferation and clonogenicity. (**A**) The cutaneous melanoma cell lines Mel Ho and SKMel28 were transfected with siRNAs targeting MAGOH, MAGOHB and MAGOH + MAGOHB and the effect on cell proliferation was monitored compared to the siCtrl for eight days by measurement with an XTT assay. The graphs represent data from three independent experiments (mean ± SD, * = *p* < 0.05, *** = *p* < 0.001, one-way ANOVA with Tukey’s Multiple Comparison Test, the stars above the two bottom lines represent both samples, siMAGOH and siMAGOH + siMAGOHB) (**B**) Summary of Absorbance measurement of the XTT assay shown in A at day 5 (mean ± SD, n = 3, * = *p* < 0.05, *** = *p* < 0.001, one-way ANOVA with Tukey’s Multiple Comparison Test). (**C**) Clonogenic assay of Mel Ho and SKMel28 after KD of MAGOH and MAGOH + MAGOHB normalized to the siCtrl. Representative pictures of one experiment are shown, graphs depict data from three replicate analyses (mean ± SD, ** = *p* < 0.01, *** = *p* < 0.001 **** = *p* < 0.0001, one-sample *t*-test compared to 1).

To investigate the importance of MAGOH and/or MAGOHB expression, respectively, for cutaneous melanoma cell proliferation, XTT proliferation assays were performed after single or combined KD of MAGOH and MAGOHB in Mel Ho and SKMel28. The results showed a significant reduction in cell proliferation after single KD of MAGOH and an even stronger effect with an almost complete inhibition of proliferation after the combined MAGOH + MAGOHB KD (Figure 3A,B). The effect was, however, smaller after KD of only MAGOHB (Figure 3A,B). 

An important property of tumor cells is the ability to form colonies derived from one single cell. This ability is tested in a clonogenic assay, which was performed with Mel Ho and SKMel28 after KD of MAGOH and/or MAGOHB. The clonogenic assay showed a strongly reduced number of colonies with the single KD of MAGOH and the simultaneous KD of MAGOH and MAGOHB when compared to the siCtrl (Figure 3B). A small but not significant reduction in colony formation was observed for the cells depleted of MAGOHB (Figure 3B). Together, the results revealed that cutaneous melanoma cells primarily rely on the expression of MAGOH, and not MAGOHB, for cell proliferation. To substantiate these results, the expression of MAGOH was knocked-down in three additional cutaneous melanoma cell lines with the use of two different MAGOH siRNAs and the effect on cell proliferation and colony formation was analyzed via XTT assay and clonogenic assay (Supplementary Figure S1A–C). These experiments additionally confirmed that the expression of MAGOH is vital for melanoma cell proliferation.

### 3.5. Knockdown of MAGOH and MAGOHB Does Not Affect Cell Cycle Progression but Induces Apoptosis

**Figure 4:**
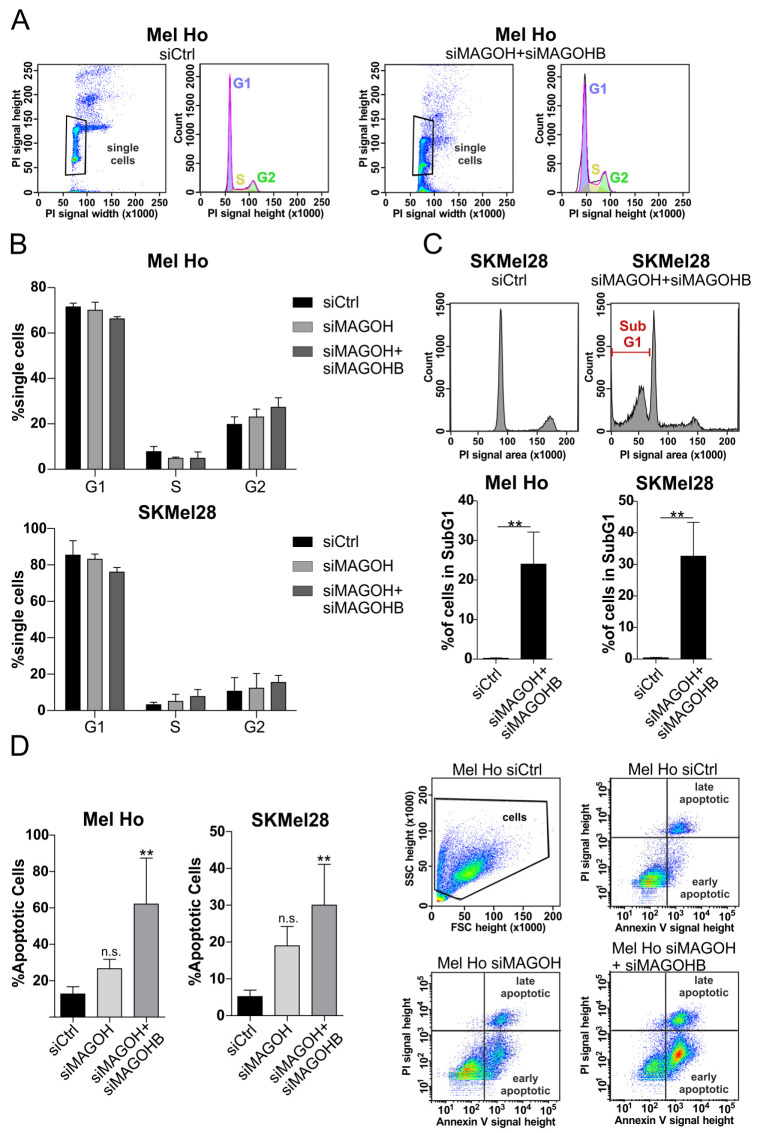
MAGOH and MAGOHB do not influence cell cycle progression, but a simultaneous knockdown of MAGOH and MAGOHB induces apoptosis in cutaneous melanoma. (**A**) Analysis of cell cycle using propidium iodide (PI) staining of the DNA content and flow cytometry. Pictures show one exemplarily experiment in Mel Ho transfected for 96 h with siCtrl or siMAGOH + siMAGOHB. Single cells were gated as depicted and analyzed with the Dean-Jett-Fox model. (**B**) Analyzed single cells (%) which have been matched to the indicated cell cycle phases as described in A. Mel Ho and SKMel28 were transfected with siCtrl, siMAGOH or siMAGOH + siMAGOHB for 96 h (mean ± SD from n = 3, one-way ANOVA with Tukey’s Multiple Comparison Test showed no significance). (**C**) Cells in the SubG1 phase, that have a decreased DNA content (lower PI signal than G1 phase) indicating cell death, were analyzed after MAGOH + MAGOHB KD for Mel Ho and SKMel28 compared to the siCtrl. Pictures represent one exemplary experiment in SkMel28, bars summarize three independent experiments (mean ± SD, ** = *p* < 0.01, unpaired *t*-test). (**D**) The occurrence of apoptotic cells (%) was measured after 96 h KD of MAGOH and MAGOH + MAGOHB in Mel Ho and SKMel28 compared to siCtrl-transfected cells using Annexin V-FITC and PI staining of living cells, followed by flow cytometry. The graphs represent data from three replicate experiments (mean ± SD, ** = *p* < 0.01, n.s. = not significant, one-way ANOVA with Tukey’s Multiple Comparison Test), the pictures indicate one exemplary staining of Mel Ho and the gating during flow cytometry.

To investigate if the observed inhibition of cell proliferation after KD of MAGOH or MAGOH + MAGOHB was due to cell cycle arrest, the siRNA-transfected cutaneous melanoma cell lines Mel Ho and SKMel28 were stained with propidium iodide (PI) and analyzed using flow cytometry (Figure 4A). Since the single KD of MAGOHB had a limited influence on cell proliferation, we concentrated the following experiments on the effect of siMAGOH and siMAGOH + siMAGOHB. The analysis showed no significant difference in the number of cells in the G0/G1, S or G2 cell cycle phases for the cells lacking MAGOH or MAGOH/MAGOHB expression when compared to the siCtrl (Figure 4B). However, a strong increase in cells in the SubG1 phase was observed after a simultaneous KD of MAGOH/MAGOHB (Figure 4C). Cells that are categorized in the SubG1 phase have a decreased DNA content compared to those in the G0/G1 phase, which is an indication of cell death. 

As an approach to further investigate the occurrence of cell death, siMAGOH- or siMAGOH + siMAGOHB-transfected, living cells were stained with fluorescent labeled Annexin V and PI and analyzed via flow cytometry to investigate apoptosis. The experiment revealed that while a single KD of MAGOH increased the number of apoptotic cells when compared to the siCtrl, this effect was only statistically significant after a combined KD of MAGOH and MAGOHB for the melanoma cell lines Mel Ho and SKMel28 (Figure 4D). Interestingly, in several other cutaneous melanoma cell lines, KD of only MAGOH was sufficient for inducing apoptosis, further indicating MAGOH as the more vital of the two homologues (Supplementary Figure S2A–C).

These results demonstrate that cutaneous malignant melanoma cells rely on the expression of MAGOH for their survival. However, for some melanoma cell lines, such as Mel Ho and SKMel28, MAGOHB also had to be knocked-down to induce a stronger and statistically significant effect on apoptosis.

In order to achieve a reliable inhibition of the cellular function of both homologues and to gain a sufficient KD effect, we designed a Pool of siRNAs targeting both MAGOH and MAGOHB. Using a siPool increases specificity and reduces off-target effects, as the concentration of each single siRNA in the Pool can be chosen to be substantially lower to gain the same KD effect as for the use of only one siRNA. The KD efficiency of the siPool on MAGOH and MAGOHB expression was confirmed in the cutaneous melanoma cell lines Mel Ho and 501Mel using qRT-PCR and Western blot analysis (Supplementary Figure S3A,B). In addition, transfection of Mel Ho and 501Mel with the siMAGOH/B Pool significantly reduced proliferation of the melanoma cells measured by XTT assay (Supplementary Figure S3C). Analyzing the occurrence of apoptosis showed that the KD of MAGOH and MAGOHB in melanoma cells using the siMAGOH/B Pool also significantly increased the proportion of apoptotic cells at a similar level compared to the simultaneous KD of MAGOH and MAGOHB with single siRNAs (Supplementary Figure S3D).

### 3.6. MAGOH and MAGOHB Do Not Regulate Alternative Splicing of the Pro-Apoptotic Gene Bcl-XS or Exon Skipping in Cutaneous Melanoma

**Figure 5:**
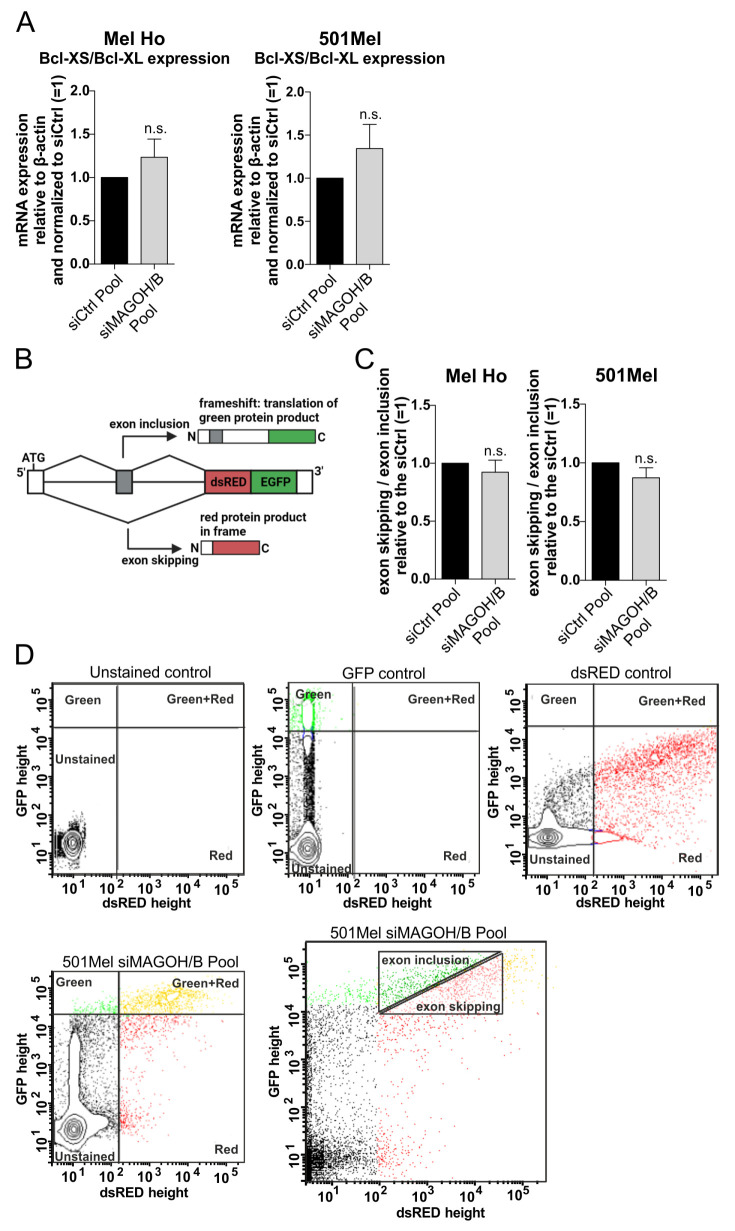
MAGOH and MAGOHB do not influence alternative splicing of pro-apoptotic Bcl-XS or exon skipping of a fluorescent splicing reporter in cutaneous malignant melanoma. (**A**) MAGOH and MAGOHB were knocked-down in Mel Ho and 501Mel for 96 h using a siPool and the mRNA levels of Bcl-XS and Bcl-XL were measured with qRT-PCR relative to β-actin (ΔCP). The ratio of Bcl-XS/Bcl-XL expression was normalized to cells transfected with the siCtrl Pool. (mean ± SD from n = 3, n.s. = not significant, one sample *t*-test compared to 1). (**B**) To analyze alternative exon usage, a reporter plasmid was utilized, which leads to the translation of a green fluorescent protein (EGFP), if an upstream exon is included during splicing of the reporter mRNA or to translation of a red fluorescent protein (dsRED), when the upstream exon is skipped. (**C**) MAGOH and MAGOHB were knocked-down in Mel Ho and 501Mel with the siMAGOH/B Pool for 24 h followed by additional 24 h transfection of the splicing reporter plasmid. Fluorescent cells were analyzed by flow cytometry. The ratio between exon skipping/exon inclusion was determined and compared to the siCtrl Pool (mean ± SD from n = 3, n.s. = not significant, one sample *t*-test compared to 1). (**D**) Exemplary pictures showing the gating used for flow cytometry of the splicing reporter analyzed in C. Transfection of plasmids expressing only dsRED or only EGFP were used as controls to set the gate for the double positive population. In cells transfected with the reporter, the heterogeneous cell population, expressing both red and green fluorescent proteins, was evaluated. For these cells, the ratio between exon inclusion and exon skipping was determined as indicated.

The previous results showed that a KD of MAGOH and MAGOHB dramatically induces apoptosis in cutaneous malignant melanoma cells. To investigate the molecular mechanism behind this effect, we analyzed if aberrations in alternative splicing caused by the loss of the two MAGOH homologues could be responsible. It is known that a KD of other core components of the EJC causes dramatic changes in alternative splicing in human cells [19]. One important example is Bcl-XS, a regulator of the intrinsic apoptotic pathway. Two different isoforms, the anti-apoptotic Bcl-XL and the pro-apoptotic Bcl-XS are produced from the same gene by the alternative usage of two competing 5′ splice sites. A loss of EJC function has been identified to increase the occurrence of the shorter Bcl-XS form and induce apoptosis [37]. To investigate if this mechanism is responsible for the observed apoptosis after KD of MAGOH and MAGOHB in melanoma cells, we transfected the siPool targeting both homologues (siMAGOH/B Pool) into the cutaneous melanoma cell lines Mel Ho and 501Mel and measured the mRNA levels of Bcl-XS and Bcl-XL using qRT-PCR. However, the experiment showed no significant effect on the Bcl-XS/Bcl-XL expression ratio for the MAGOH/B KD cells when compared to the siCtrl Pool, indicating no increase in alternative splicing of the pro-apoptotic Bcl-XS variant (Figure 5A).

Continuing, we wanted to investigate a possible influence of the MAGOH/B KD on alternative splicing in a more general, gene independent manner. Therefore, the expression of both MAGOH and MAGOHB was knocked-down in Mel Ho and 501Mel with the siMAGOH/B Pool, followed by transfection with a fluorescent splicing reporter. This reporter contains an exon between two intronic sequences followed by a dsRED/EGFP cassette (Figure 5B) [30]. In the case of exon inclusion, a frameshift causes translation of only EGFP while exon skipping leads to the production of dsRED. As controls, plasmids constitutively expressing only EGFP or only dsRED were used. The experiment showed that a KD of MAGOH/B did not lead to a shift to neither more exon skipping nor more exon inclusion in Mel Ho or 501Mel (Figure 5C,D) indicating that MAGOH and MAGOHB do not influence this kind of exon skipping in cutaneous melanoma.

Consistent with the fact that the MAGOH/B KD had no effect on alternative splicing of the Bcl gene, the regulation of splicing seems to play a minor role in the function of MAGOH and MAGOHB in cutaneous malignant melanoma.

### 3.7. MAGOH and MAGOHB Knockdown Decreases NMD Activity and Promotes Apoptosis via Upregulation of GADD45A

**Figure 6:**
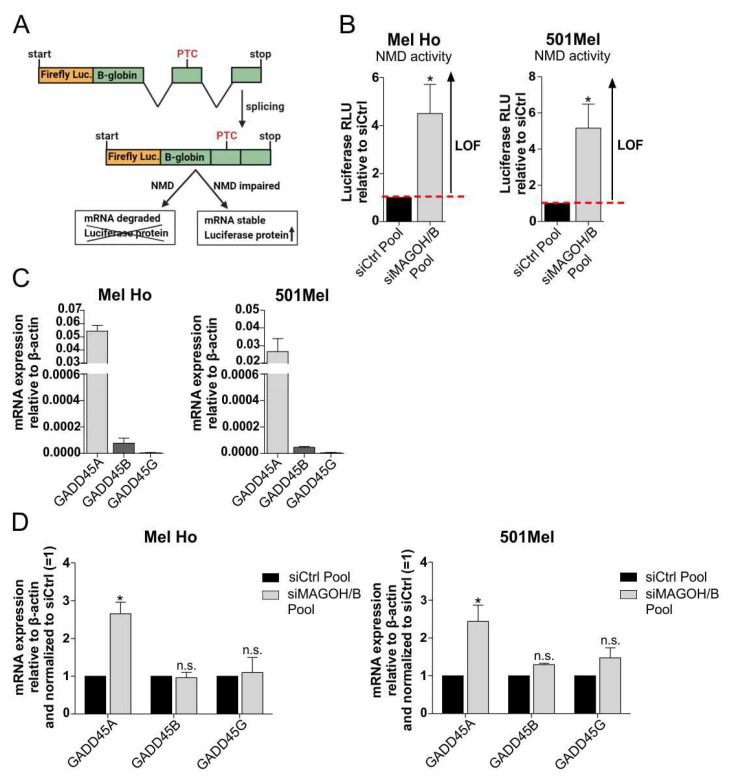
MAGOH and MAGOHB influence NMD activity and the expression of GADD45A in cutaneous malignant melanoma. (**A**) The NMD reporter plasmid encodes a firefly luciferase (Luc.), fused to a PTC-containing β-globin sequence, inducing degradation of the luciferase mRNA via NMD. A loss of function (LOF) of NMD impairs sensing of the PTC, leading to an increased luciferase signal. (**B**) MAGOH and MAGOHB were knocked-down in Mel Ho and 501Mel using the siMAGOH/B Pool for 24 h, followed by another 24 h transfection with the NMD reporter or the control plasmid. Luciferase relative light units (RLU) were calculated as (average luciferase/renilla NMD reporter)/(average luciferase/renilla control plasmid) and normalized to the siCtrl Pool (mean ± SD from n = 3, * = *p* < 0.05, one sample *t*-test compared to 1). (**C**) MRNA expression of GADD45A, GADD45B and GADD45G was analyzed relative to β-actin (ΔCP) with qRT-PCR in Mel Ho and 501Mel (mean ± SD from n = 3). (**D**) MRNA expression of GADD45A, GADD45B and GADD45G after KD of MAGOH/B for 48 h was investigated with qRT-PCR relative to β-actin (ΔCP) and was normalized to the siCtrl Pool (mean ± SD from n = 3, * = *p* < 0.05, n.s. = not significant, one sample *t*-test compared to 1).

Alongside alternative splicing, the EJC is also involved in the cellular process of NMD. The NMD pathway plays an important role in the regulation of cell death, as the over-reading of PTCs leads to the production of toxic protein forms that can trigger apoptosis [16,17,38]. In order to assess if MAGOH and MAGOHB influence NMD activity in cutaneous melanoma, their expression was knocked-down in Mel Ho and 501Mel using the siMAGOH/B Pool and the cells were subsequently transfected with a NMD reporter plasmid. The reporter contains the coding sequence of the firefly luciferase fused in frame to a PTC-containing β-globin sequence (Figure 6A) [31]. A loss of NMD function would stop the degradation of the PTC containing construct, consequently accumulating the luciferase signal in the cells. As control, cells were transfected with a comparable luciferase-β-globin construct, but without a PTC. The experiment revealed that KD of MAGOH/B significantly increased the luciferase relative light units (RLU) for Mel Ho and 501Mel compared to the siCtrl Pool-transfected cells (Figure 6B), indicating a loss of NMD function (LOF). These results demonstrate that cutaneous melanoma cells rely on the expression of MAGOH and MAGOHB for NMD pathway activity.

To connect the decreased NMD activity after MAGOH/B KD to the observed apoptotic phenotype in melanoma cells, we further investigated GADD45. GADD45 is a pro-apoptotic protein which has previously been reported to be regulated by the process of NMD [16,38]. In human cells, three variants of the GADD45 mRNA occur: GADD45A, GADD45B and GADD45G. To investigate which variants might play a role in cutaneous melanoma cells, the general expression was analyzed in Mel Ho and 501Mel via qRT-PCR. The data show that GADD46G is not expressed in both cell lines, whereas GADD45A mRNA is the most abundant isoform (Figure 6C). 

After KD of MAGOH and MAGOHB using the siMAGOH/B Pool, a significant increase in GADD45A mRNA expression was observed in the cutaneous melanoma cell lines Mel Ho and 501Mel, whereas no obvious changes in the expression of GADD45B or GADD45G could be detected (Figure 6D). This indicates a significant influence of MAGOH and MAGOHB on the expression of the pro-apoptotic NMD target GADD45A in cutaneous malignant melanoma.

**Figure 7:**
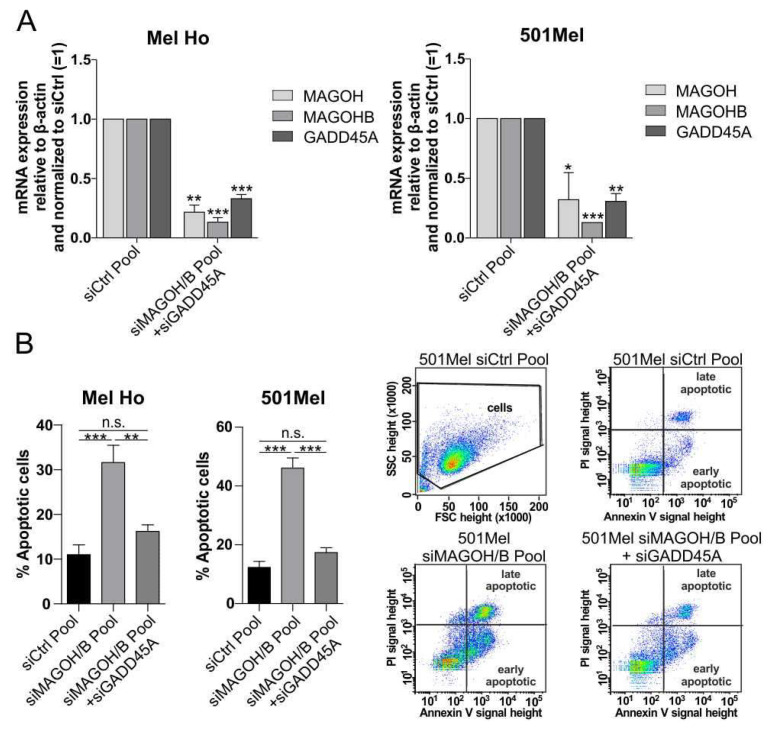
Simultaneous knockdown of GADD45A and MAGOH/B in cutaneous melanoma cell lines significantly rescues the cells from apoptosis. (**A**) MRNA expression of MAGOH, MAGOHB and GADD45A after simultaneous transfection of Mel Ho or 501Mel for 48 h with the siMAGOH/B Pool and a siRNA targeting GADD45A relative to β-actin (ΔCP) and normalized to the siCtrl Pool analyzed with qRT-PCR (mean ± SD from n = 3, * = *p* < 0.05, ** = *p* < 0.01 *** = *p* < 0.001, one sample *t*-test compared to 1). (**B**) The occurrence of apoptotic cells (%) was measured after 72 h of co-transfection with the siMAGOH/B Pool and a siRNA targeting GADD45A compared to the siCtrl Pool, by staining of living cells with PI and Annexin V-FITC, followed by flow cytometry analysis. The graphs represent data from three replicate experiments (mean ± SD, ** = *p* < 0.01, *** = *p* < 0.001, n.s. = not significant, one-way ANOVA with Tukey’s Multiple Comparison Test), the pictures indicate one exemplary staining of 501Mel and the gating during flow cytometry.

To analyze how the increased GADD45A expression after MAGOH and MAGOHB deficiency in cutaneous melanoma cells influences the observed apoptotic phenotype, siMAGOH/B Pool-transfected melanoma cells were additionally transfected with a siRNA targeting GADD45A. Thereby, the increased GADD45A expression observed after the loss of MAGOH/B could be efficiently downregulated in the cutaneous melanoma cell lines Mel Ho and 501Mel (Figure 7A). Furthermore, additionally knocking down GADD45A expression after KD of MAGOH/B significantly decreased the occurrence of apoptosis in melanoma cells (Figure 7B), indicating that the increased GADD45A expression might be one of the main reasons behind the apoptosis observed after loss of MAGOH and MAGOHB expression in cutaneous malignant melanoma cells.

## 4. Discussion

Cancer progression and tumorigenesis is induced by an accelerated cell division rate caused e.g., by an inactivation of apoptosis inducing pathways. During tumorigenesis, tumors exhibit a positive selection for cells harboring favorable characteristics for cell growth and survival. This can, for example, manifest through a clonal expansion of cancer cells which display an upregulated expression of proteins essential for these pathological processes [39]. 

This study investigates the influence of MAGOH and its highly identical homologue MAGOHB with regards to cutaneous melanoma pathogenesis and cancer progression. MAGOH, as a core component of the EJC, regulates several aspects of gene expression [14]. 

We identified that several cutaneous melanoma cell lines have an upregulated protein level of MAGOH and MAGOHB when compared to normal human epidermal melanocytes. A high protein expression of MAGOH and MAGOHB was also demonstrated for patient derived cutaneous melanoma tumors in vivo. This is a first indication that the expression of these two homologous proteins is favorable for melanoma pathogenesis. 

This study revealed that cutaneous melanoma cells express significantly more MAGOH compared to MAGOHB on the mRNA as well as on the protein level. Using functional assays, we could show that MAGOH, and not MAGOHB, is vital for melanoma cell growth, as an siRNA-mediated KD of only MAGOH significantly inhibits cell proliferation. In most cutaneous melanoma cell lines, the KD of MAGOH is also sufficient to significantly induce apoptosis, suggesting that the reduced proliferation is mainly caused by the occurrence of cell death. 

It was previously shown that MAGOH and MAGOHB can form the EJC complex to the same extend [20]. Therefore, it can be argued that the limited influence of a KD of MAGOHB, observed in the proliferation and viability assays, was due to the persisting expression of MAGOH in these cells. At the same time, the low remaining expression of MAGOHB in the MAGOH KD cells was insufficient to maintain an essential level of EJC activity. These results indicate that, despite a functional redundance of both homologues in the EJC, MAGOH is the predominant and more essential homologue for melanoma cell viability. 

Previous studies have identified that e.g., in gastric cancer, a depletion of MAGOH or MAGOH and MAGOHB, next to inducing apoptosis, also significantly inhibited cell cycle progression [24]. Interestingly, no significant effect on cell cycle phases was observed after MAGOH or MAGOH/MAGOHB KD for our melanoma cell lines. This could mean that, compared to other cancer types, cutaneous melanoma exhibits an increased sensitivity to MAGOH (and MAGOHB) depletion with regards to apoptosis, meaning that cutaneous malignant melanoma cells undergo apoptosis before the cell cycle is affected. 

In this study, analyzing the influence of MAGOH and MAGOHB on cell proliferation and apoptosis, we observed additive effects after a simultaneous depletion of both homologues in cutaneous melanoma cells. Similar results have previously been described for other types of cancer where both MAGOH and MAGOHB had to be depleted to gain a sufficient KD of MAGOH/MAGOHB protein levels to interrupt EJC function and to reduce tumor cell viability as well as Xenograft tumor growth in mice [20,22,24]. Considering their high sequence identity and same level of affinity to form the EJC complex [20], in the case to utilize MAGOH as a therapeutic target, it would be reasonable to develop an inhibitor which targets both MAGOH and MAGOHB. To achieve an efficient, simultaneous KD of both homologues and to gain a sufficient effect on cutaneous malignant melanoma cells, we designed a Pool of siRNAs targeting both MAGOH and MAGOHB. Through this approach, we ensured that no functional effects of the KD were missed because of possible compensations from the persisting expression of the other homologue. Using the siMAGOH/B Pool we obtained an efficient KD of both homologues, leading to the impaired proliferation of melanoma cells and the induction of apoptosis. This strategy could be the basis for a future therapeutic approach to impair the growth of cutaneous melanoma cells.

Until now, the functional influence of MAGOH and MAGOHB in cancer has been essentially unexplored. Investigating the molecular mechanisms behind the observed cell death after KD of MAGOH and MAGOHB, we revealed a significant influence on the mechanism of NMD in cutaneous melanoma. 

Previous research has identified that NMD can play an important role in cancer progression [40]. However, the way tumor cells use the NMD mechanism for promoting cell growth or escaping cell death is highly complex depending on the type of tumor and its genetic evolution [40]. In some cancers, such as pancreatic adenosquamous carcinoma or inflammatory myofibroblastic tumors, inactivating mutations in NMD factors have been identified; they promote an upregulation of important oncogenes that are normally under tight regulation of the NMD process in human cells [40]. In several other types of tumors, PTCs have been found to be enriched in tumor suppressor genes, being able to potentiate tumorigenesis via the NMD mechanism [16]. In this study, we demonstrate that MAGOH and MAGOHB play an essential role in maintaining NMD activity in cutaneous melanoma. The KD of both MAGOH homologues leads to a loss of NMD function in cutaneous melanoma cells and induces apoptosis. This indicates a tumor promoting function of NMD in melanoma which could, according to the mechanism in other tumor types, be mediated by an NMD-induced downregulation of tumor suppressor genes. In this study we could identify the NMD target GADD45A as strongly and significantly upregulated in cutaneous melanoma cells after KD of MAGOH and MAGOHB. In addition, it was further shown that a simultaneous downregulation of GADD45A together with MAGOH and MAGOHB could attenuate cell death in cutaneous melanoma cells, indicating GADD45A a strong tumor suppressor in cutaneous malignant melanoma.

The protein GADD45A (Growth arrest and DNA damage-inducible 45 A) is involved in the cellular DNA damage response pathway and serves as a stress sensor [41]. The expression of the GADD45 proteins can be regulated by numerous cellular pathways, which also play an important role in cancer, such as the mitogen activated protein kinases JNK and p38, the tumor suppressor p53 or the transcription factors FOXOA3 and ATF4 [42]. A constitutive activation of the ERK kinase, which can be frequently found in melanoma tumors, leads to a downregulation of GADD45A [42]. The regulation of GADD45A by the NMD pathway has so far been shown in Drosophila and mouse embryonic fibroblasts [38]. Our study demonstrates that the KD of MAGOH and MAGOHB increased GADD45A expression in cutaneous melanoma, an effect that might be mediated by an impairment of NMD induced by the loss of MAGOH and MAGOHB.

By its involvement in the DNA damage repair pathway, GADD45A can play an important role in the response to cancer chemotherapies that cause DNA damage, such as the DNA alkylating agent Cisplatin. In melanoma, it has been shown by Liu et al. that treatment with Cisplatin increases GADD45A expression and a KD of GADD45A could enhance the reduced proliferation caused by the Cisplatin treatment [26]. The authors of the study conclude that GADD45A could play a role in the resistance to classical chemotherapies often occurring in melanoma through a hyperactivation of the DNA damage response [26]. In our study we observed an increase in apoptosis caused by an upregulation of GADD45A and that a KD of GADD45A attenuated cell death. As this increase in GADD45A is caused by a loss of NMD regulation of GADD45A following MAGOH and MAGOHB KD, it is supposed to be independent of DNA damage. A KD of MAGOH/MAGOHB and the resulting increase in GADD45A expression in cutaneous melanoma could possibly enhance the effect of other cancer therapies whose main mode of action is not to induce DNA damage, such as targeted therapy or immunotherapy. It has been shown that NMD escape mutations can positively influence the benefit of melanoma patients from immune checkpoint inhibitors or adoptive T cell therapy [43]. Considering this, an inhibition of NMD, as performed in this study via MAGOH/MAGOHB KD, could have a comparable effect. 

Our findings demonstrate that cutaneous malignant melanoma cells rely on the expression of primarily MAGOH, but to an extent also MAGOHB, for their survival. A simultaneous inhibition of MAGOH and MAGOHB could be an approach to trigger melanoma cells to cell death, possibly enhance effects of other therapies and reduce cancer progression. Hence, the inhibition of MAGOH and MAGOHB could constitute a promising new approach for the treatment of cutaneous melanoma.

## Figures and Tables

**Table 1:** Human cutaneous melanoma cell lines.

Cell Line	Origin	Medium	CO_2_	ID
Sbcl2	PT ^1^	TU	5%	RRID:CVCL_D732
Mel Ho	PT	RPM1-1640	8%	RRID:CVCL_1402
WM1366	PT	TU	5%	RRID:CVCL_6789
WM3211	PT	TU	5%	RRID:CVCL_6797
501Mel	MET ^2^	RPMI-1640	8%	RRID:CVCL_4633
SKMel28	MET	DMEM	8%	RRID:CVCL_0526
WM1158	MET	TU	5%	RRID:CVCL_6785

^1^ Derived from melanoma primary tumor of the skin; ^2^ Derived from cutaneous melanoma metastasis.

**Table 2:** SiRNA sequences.

siRNA	Sequence (5′-3′)	Manufacturer
siMAGOH	GCGUGAUGGAGGAACUGAATT ^1^	Sigma-Aldrich Chemie GmbH, Steinheim, Germany
siMAGOH2	“Hs_MAGOH_8 FlexiTube siRNA” GeneGlobe ID: SI05064101	QIAGEN GmbH, Hilden, Germany
siMAGOHB	GGCUGUUUGUAUAUUUAAUTT ^1^	Sigma-Aldrich Chemie GmbH, Steinheim, Germany
siCtrl	“AllStars Negative Control siRNA”	QIAGEN GmbH, Hilden, Germany
siGADD45A	GGAGGAAGUGCUAGCAAATT ^2^	Sigma-Aldrich Chemie GmbH, Steinheim, Germany
siMAGOH/B Pool	Mixture of 30 different siRNAs targeting MAGOH and MAGOHB	siTools Biotech, Planegg, Germany
siCtrl Pool	Mixture of 30 non-targeting siRNAs	siTools Biotech, Planegg, Germany

^1^ siRNA sequences from Zhou et al. [24]; ^2^ siRNA sequence from Liu et al. [26].

**Table 3:** QRT-PCR primer sequences and temperatures for measurement.

Primer Name	Sequence (5′-3′)	Measurement (°C)
hBcl-x 1095 rev	GGGAGGGTAGAGTGGATGGT	≤85
hBcl-XL 887 fwd	TGACCACCTAGAGCCTTGGA	≤85
hBcl-Xs 749 fwd	TGAACAGGATACTTTTGTGGAACT	≤85
Beta actin_cyto fwd	CTACGTCGCCCTGGACTTCGAGC	≤85
Beta-Actin_cyto rev	GATGGAGCCGCCGATCCACACGG	≤85
hGADD45A 399 fwd	ATCACTGTCGGGGTGTACGA	≤86
hGADD45A 619 rev	CAGCGTCGGTCTCCAAGA	≤86
hGADD45B 443 fwd	TGCAAATCCACTTCACGCTC	≤88
hGADD45B 613 rev	CGTGTGAGGGTTCGTGACC	≤88
hGADD56G 235 fwd	CGTCTACGAGTCAGCCAAAGT	≤88
hGADD45G 394 rev	ACGCGCACTATGTCGATGT	≤88
hMAGOH 150 fwd	GACCGGACGGGAAGTTAAGA	≤75
hMAGOH 251 rev	TCAGTTCCTCCATCACGCTTT	≤75
hMAGOHB 106 fwd	GCCGGACGGAAAGCTTAGAT	≤75
hMAGOHB 341 rev	GGCCTTCAGGATCCTTTGACT	≤75

## Data Availability

The data presented in this study are available on request from the corresponding author.

## Supplementary Materials

The following supporting information can be downloaded at: https://www.mdpi.com/article/10.3390/cells11233859/s1, Supplementary Figure S1: Knockdown of MAGOH with two different siRNAs inhibits cell proliferation of the melanoma cell lines WM1158, 501Mel and Sbcl2; Supplementary Figure S2: Knockdown of MAGOH with two different siRNAs induces apoptosis in the melanoma cell lines WM1158, Sbcl2 and 501Mel; Supplementary Figure S3: Assessment of the efficiency and influence of a siRNA Pool designed to simultaneously knock down the expression of MAGOH and MAGOHB showed comparable results to the treatment with the single MAGOH and MAGOHB siRNAs.
