# Supplementary material for: MAGOH and MAGOHB Knockdown in Melanoma Cells Decreases Nonsense-Mediated Decay Activity and Promotes Apoptosis via Upregulation of GADD45A
Source: Cells. 2022 Nov 30;11(23):3859. doi: 10.3390/cells11233859 (PMC9738831; doi:10.3390/cells11233859)
Supplement: Supplementary file 1 [file cells-11-03859-s001.zip › cells-2026591-supplementary.pdf]

Supplementary File 1

Supplementary Figures S1-S3

MAGOH and MAGOHB knockdown in melanoma cells decreases nonsense-mediated decay activity and promotes apoptosis via upregulation of GADD45A

Agnes Soederberg, Tina Meißgeier, Anja Katrin Bosserhoff and Lisa Linck-Paulus

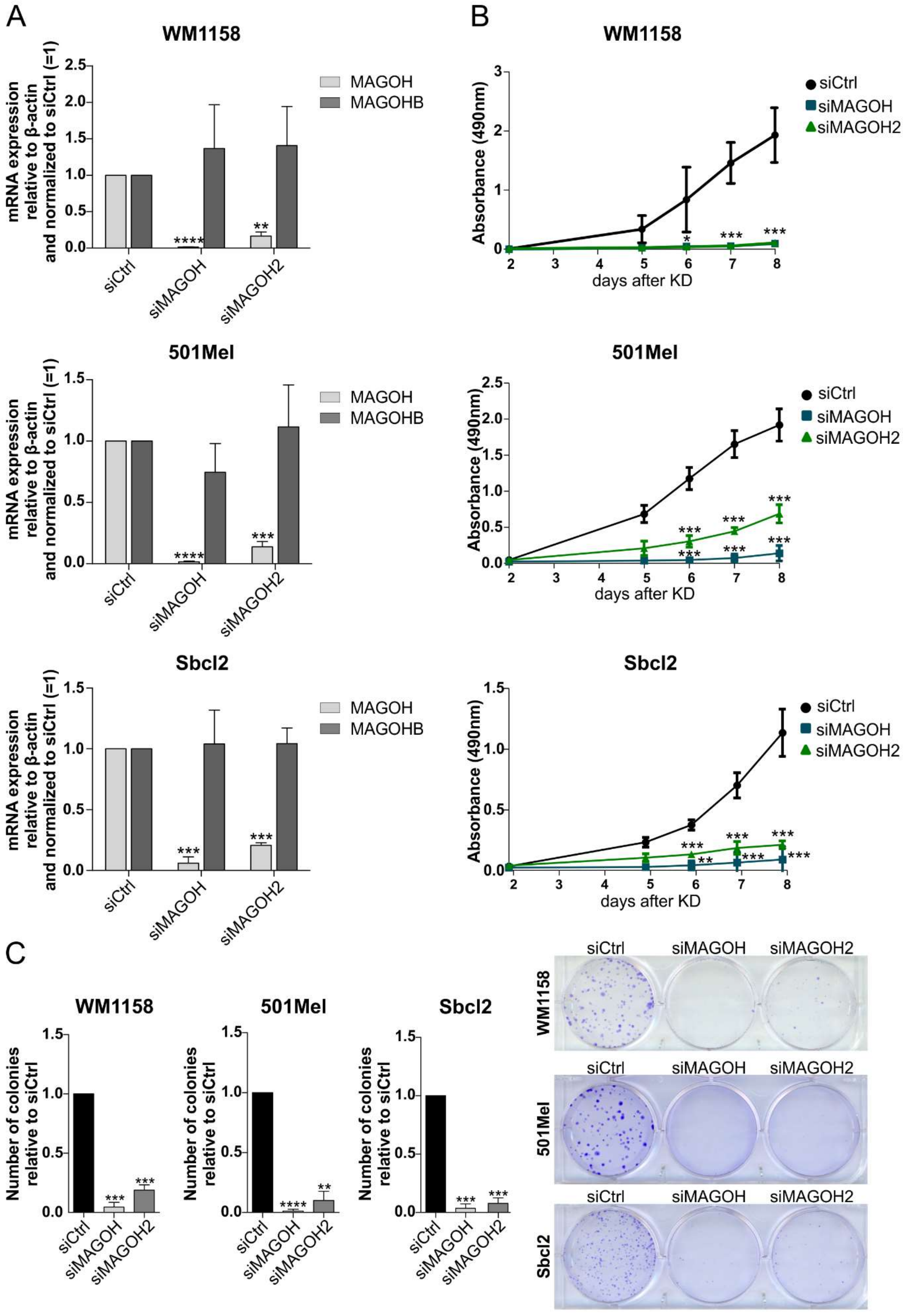

Supplementary Figure S1: Knockdown of MAGOH with two different siRNAs inhibits cell proliferation of the melanoma cell lines WM1158, 501Mel and Sbcl2. (**A**) The melanoma cell lines WM1158, 501Mel and Sbcl2 were transfected with two different siRNAs targeting MAGOH and the KD efficiency as well as potential off-target effects on MAGOHB expression were evaluated with qRT-PCR relative to β-actin (ΔCP) and normalized to

a control siRNA (mean ± SD from n=3, **=$p < 0.01$ ***=$p < 0.001$, ****=$p < 0.0001$, one sample t-test compared to 1). (**B**) The expression of MAGOH was knocked down with two different siRNAs in the melanoma cell lines WM1158, 501Mel and Sbcl2 and the effect on cell proliferation was monitored compared to the siCtrl for seven days by measurement with an XTT assay. The graphs represent data from three replicate experiments (mean ± SD, *=$p < 0.05$, ***=$p < 0.001$, One-way ANOVA with Tukey's Multiple Comparison Test post-test Tukey). (**C**) The expression of MAGOH was knocked down in WM1158, 501Mel and Sbcl2, using two different siRNAs, and the effect on colony formation was analyzed with a clonogenic assay normalized to the siCtrl. The graphs represent data from three replicate analyses (mean ± SD, **=$p < 0.01$, ***=$p < 0.001$ ****=$p < 0.0001$, one-sample t-test compared to 1), pictures show one representative experiment.

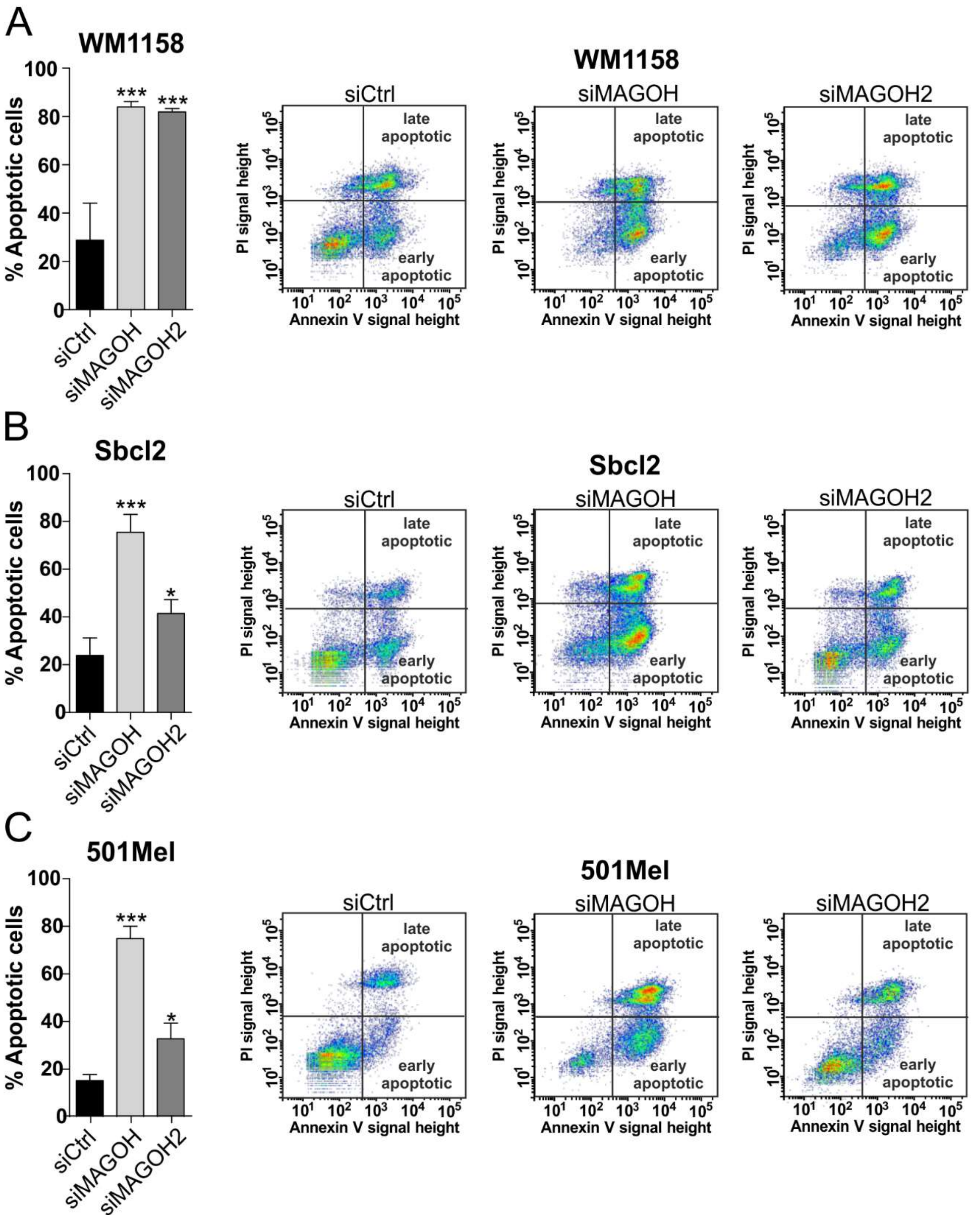

Supplementary Figure S2: Knockdown of MAGOH with two different siRNAs induces apoptosis in the melanoma cell lines WM1158, Sbcl2 and 501Mel. Following 96h of MAGOH KD using two different siRNAs, the occurrence of apoptotic cells (%) was measured compared to the siCtrl for (**A**) WM1158, (**B**) Sbcl2 and (**C**) 501Mel by staining living cells with PI and Annexin V-FITC followed by analysis with flow cytometry. The pictures show one exemplary staining and the gating for flow cytometry. The graphs represent data form three replicate analyses (mean ± SD, *=$p < 0.05$, ***=$p < 0.001$, one way ANOVA with Tukey's Multiple Comparison Test).

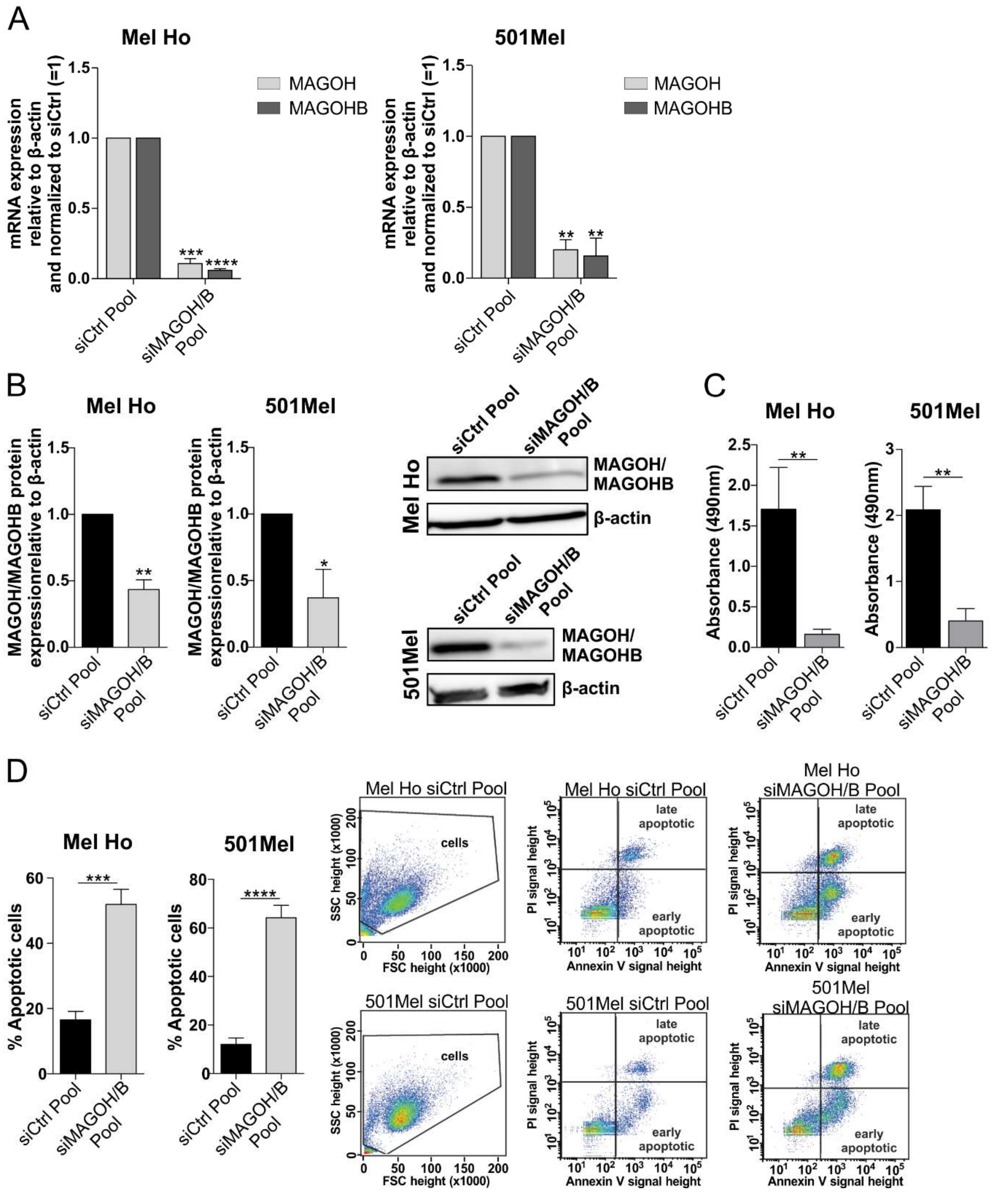

Supplementary Figure S3: Assessment of the efficiency and influence of a siRNA Pool designed to simultaneously knock down the expression of MAGOH and MAGOHB showed comparable results to the treatment with the single MAGOH and MAGOHB siRNAs. (**A**) MRNA expression of MAGOH and MAGOHB in Mel Ho and 501Mel after 48h transfection with the siMAGOH/B Pool relative to β-actin (ΔCP) and normalized to the siCtrl Pool was analyzed with qRT-PCR (mean ± SD from n=3, **=p < 0.01 ***=p < 0.001, ****=p < 0.0001, one sample t-test compared to 1). (**B**) Western blot analysis of MAGOH/MAGOHB protein expression after 48h KD with the siMAGOH/B Pool compared to the siCtrl Pool (mean ± SD from n=3, *=p<0.05, **=p < 0.01, one sample t-test compared to 1). Pictures represent one exemplary experiment. (**C**) MAGOH and MAGOHB were knocked down in Mel Ho and 501Mel using the siMAGOH/B Pool and the influence on cell proliferation measured with an XTT assay. The graph represents the measurement after seven days of transfection compared to the siCtrl (mean ± SD from n=3, **=p < 0.01, unpaired t-test). (**D**) The occurrence of apoptotic cells (%) was measured after 96h of transfection with the siMAGOH/B Pool compared to the siCtrl Pool, by staining living cells with PI and Annexin V-FITC followed by flow cytometry analysis.

The graphs represent data form three replicate analyses (mean ± SD, ***=$p < 0.001$, ****=$p < 0.0001$, unpaired t-test), the pictures show one exemplary experiment and the gating for flow cytometry.
